# Anomalous diffusion and q-Weibull velocity distributions in epithelial cell migration

**DOI:** 10.1371/journal.pone.0180777

**Published:** 2017-07-10

**Authors:** Tatiane Souza Vilela Podestá, Tiago Venzel Rosembach, Anésia Aparecida dos Santos, Marcelo Lobato Martins

**Affiliations:** 1 Departamento de Física, Universidade Federal de Viçosa, Viçosa, Minas Gerais, Brazil; 2 Departamento de Biologia Geral, Universidade Federal de Viçosa, Viçosa, Minas Gerais, Brazil; 3 National Institute of Science and Technology for Complex Systems, Brazil; Semmelweis Egyetem, HUNGARY

## Abstract

In multicellular organisms, cell motility is central in all morphogenetic processes, tissue maintenance, wound healing and immune surveillance. Hence, the control of cell motion is a major demand in the creation of artificial tissues and organs. Here, cell migration assays on plastic 2D surfaces involving normal (MDCK) and tumoral (B16F10) epithelial cell lines were performed varying the initial density of plated cells. Through time-lapse microscopy quantities such as speed distributions, velocity autocorrelations and spatial correlations, as well as the scaling of mean-squared displacements were determined. We find that these cells exhibit anomalous diffusion with q-Weibull speed distributions that evolves non-monotonically to a Maxwellian distribution as the initial density of plated cells increases. Although short-ranged spatial velocity correlations mark the formation of small cell clusters, the emergence of collective motion was not observed. Finally, simulational results from a correlated random walk and the Vicsek model of collective dynamics evidence that fluctuations in cell velocity orientations are sufficient to produce q-Weibull speed distributions seen in our migration assays.

## Introduction

Cell migration is a dynamic and complex process guided by a vast array of chemical and physical signals [1]. All nucleated cell types migrate at least during a given period of their development. In multicellular organisms, the regulation of cell motility is central in all morphogenetic processes, tissue maintenance, wound healing and immune surveillance [2]. Its failure potentiates numerous diseases, including inflammation, cardiovascular disease, cancer metastasis and various birth defects [3]. On the other hand, the major goal of regenerative medicine, which is the creation of artificial tissues and organs through the colonization of biomaterials by cells, requires the control of their organization, communication and movements [4]. So, it is imperative to characterize how cells move *in vitro* and *in vivo* to understand the mechanisms that govern cell motile behavior.

Besides their biological relevance, motile cells constitute a preeminent topic of interest in the physics of active systems [5, 6]. The self-propelled, stochastic and nonlinear motions of active interacting particles generate collective dynamics, patterns on mesoscopic scales, and unusual mechanical and rheological properties that are fundamentally different from those observed in ordinary matter (purely passive gases, liquids or solids) at thermal equilibrium. Understanding the general principles that enable active agents to generate internal forces that control and direct their own motion and that of their surroundings is imperative to develop not only a theoretical description of living matter, but also a framework for a systematic engineering of biomimetic materials exhibiting actively driven far-from-equilibrium propeties.

From the statistical physicist stand point, individual cell migration can be mapped on a search process for targets (e. g., nutrients, growth factors and chemokines), which can be detected by the cell only within a limited spatial range [7]. In the absence of external gradients of such cues, motile cells perform random walks whose characteristic features probably reflect some of the molecular and subcellular mechanisms that regulate their migration phenotype. Hence, the systematic analysis of experimental time series for trajectories of migrating cells will yield much quantitative information for generate cell-type specific motility models. These “macroscopic” models of cellular behavior integrated with “microscopic” descriptions of the dynamics involving integrin binding to extracellular ligands, actin polymerization and generation of traction forces by myosin II [8] will constitute the systems biology of cell motility.

In this context, the movement of several cell types, from unicellular to multicellular organisms, were characterized. The data confirm that most cells diffuse anomalously. Indeed, cells commonly migrate with a directional persistence generating correlated random walk patterns [9]. This is the case, for instance, of *Dictyostelium* [10, 11], Hydra [12], and human mammary epithelial [13] cells, fibroblasts and keratinocytes [14]. Some of these cells [10, 14, 15] exhibit non-Gaussian speed distributions, in contrast to the Ornstein-Uhlenbeck process [16], maybe the simplest and most popular model for persistent random walks. Furthermore, micro-organisms and cells of the immune system can perform Lévy walks, a special case of superdiffusion in which the distribution of step lengths has infinite variance [17]. So, for example, the dinoflagellate *Oxyrrhis marina* executes Lévy flights when its prey decreases in abundance [18]. Also, the movement of CD8^+^ T cells in the brains of mice infected by *Toxoplasma gondii* is well described by an intermittent Lévy walk [19]. However, T and B cells migrate within intact lymph nodes by a normal random walk [20]. Summarizing, the motion of cells is rich in variety and no single universal search strategy fits to all cell types and environmental conditions.

In the present paper, we performed individual cell migration assays on plastic 2D surfaces using normal and tumoral epithelial cells plated at distinct initial densities and in conditions free from any biasing cues. Experimental time series for trajectories of migrating cells were recorded by time-lapse microscopy. We then determined speed, displacement, and turn angle distributions within these trajectories, as well as velocity autocorrelation functions. We also examined from spatial velocity correlations whether these cells execute exhibit neat cues for collective motion.

## Materials and methods

### Cell culture

Madin-Darby canine kidney (MDCK) cells, an immortalized epithelial cell line (Cell Bank, Rio de Janeiro Federal University, Rio de Janeiro, RJ, Brazil) and B16F10 cells, derived from a murine melanoma (Pharmacology Department, Minas Gerais Federal University, Belo Horizonte, MG, Brazil) were used. These cells were cultured in 25 cm^2^, 60 ml flasks (Techno Plastic Products AG 90025) at 37°C with 5% of CO_2_ in Dulbecco’s Minimum Essential Medium (high glucose Sigma Aldrich) at 7 − 7.2 pH, supplemented with 10% fetal calf serum (Cultilab, Campinas, SP, Brazil), 100 i.u./ml penicilin, 100 *μ*g/ml streptomycin, 2.5 ng/ml amphoterycin B, and 262 mM sodium bicarbonate.

Cells were sparsely seeded on either the plastic surface growth of the flasks or on the glass bottom of *μ*-Dish^35*mm*,*high*^ plates (Ibid). Distinct numbers *N* = 500, 2500, 50,000, and 250,000 cells, corresponding to densities of 20, 100, 2000, and 10,000 cells per cm^2^ were plated on the plastic surfaces. In turn, *N* = 5000 and 50,000 cells were plated on the glass surfaces, corresponding to densities of 1190 and 11,900 cells per cm^2^. All migration assays had 3 biological replicates and were performed without any externally established chemo-attractant gradients.

### Time lapse microscopy

Cell displacements were tracked via an inverted Nikon TS 100 phase-contrast microscope equipped with a CCD camera (JAI CM 140 GE) and a 10× 0.3 NA objective. Data were collected at a resolution of 1 pixel = 0.48 *μ*m^2^ from a fixed imaged field with 1392 × 1040 pixels. Imaging started 8 hours after cell plating with a video-microscopy sampling interval of 1 min and typically last for 6 h. The 8 h period ensures complete cell adhesion to the plastic substrate. Only cells that did not adhere to other cells, or undergone division or death or moved out of the imaged field were included in the analysis. The number of cells filtered for tracking procedure was at least *N* = 10 for every cell line and initial densities plated.

Large-field-of-view images for investigate possible collective cell migration were obtained by stitching together 30 fields acquired with 20× objectives using a Nikon IMq-Biostation and *μ*-Dish^35*mm*,*high*^ plates. The size of a large-field was roughly 2511 × 1557 *μ*m, corresponding to a resolution of 3840 × 2400 pixels (1 pixel = 2.33 *μ*m^2^). Typically, around 50 and 430 cells were observed per large-field at the densities tested, namely, 119 and 11,900 cells per cm^2^. The time between two successive images of these large-fields was set to 5 min. and the experiments last for 12 h.

### Data analysis

For each cell trajectory $\vec{r}\left(t\right)$, the positions ${\vec{r}}_{i}$ of the cell centroid at the times *t*_*i*_ = *i*Δ*t* (*i* = 1, 2, …, 360), were recorded. The corresponding velocities were calculated as ${\vec{v}}_{i}=\left({\vec{r}}_{i}-{\vec{r}}_{i-1}\right)/\Delta t$. From these data, velocity distributions and autocorrelation functions, as well as the probability distributions of the turn angles within cell trajectories were determined.

The speed distribution *p*(*v*) was defined as the fraction of velocity data points with speed *v* binned between *k*Δ*v* and Δ*v* = (*v*_*max*_ − *v*_*min*_)/*k*_*max*_, where *k* was varied from 0, 1, …, *k*_*max*_. As in reference [21], *k*_*max*_ = 30 was empirically chosen aiming to generate the largest number of bins, each one containing statistically significant sample of data points. Then, the distribution *p*(*v*) was built as a histogram with a fixed bin size Δ*v* = (*v*_*max*_ − *v*_*min*_)/30. For comparison, distinct values for *k*_*max*_ were tested (see S1 PDF).

The velocity autocorrelation function defined as

$$\begin{array}{c} c_{v}\left(\tau \right)=\frac{1}{N-k}\;\sum_{i=1}^{N-k}\;\frac{\vec{v}\left(t_{i}+\tau \right)\cdot \vec{v}\left(t_{i}\right)}{\left\langle \vec{v}{\left(t\right)}^{2}\right\rangle } \end{array}$$ (1)

where *N* is the total number of data points and *k* = *τ*/Δ*t* is the number of sampling intervals associated to the time lapse *τ* considered. As in references [10] and [21], *c*_*v*_(*τ*) was calculated for each trajectory as

$$\begin{array}{c} c_{v}\left(\tau \right)=\frac{\frac{1}{N-\tau -1}\;\sum_{i=1}^{N-\tau }\;\left({\vec{v}}_{i}-\frac{1}{N-\tau }{\;\sum }_{j=1}^{N-\tau }\;{\vec{v}}_{j}\right)\;\cdot \;\left({\vec{v}}_{i+\tau }\;-\;\frac{1}{N-\tau }\;\sum_{j=\tau +1}^{N}\;{\vec{v}}_{j}\right)}{\left[\frac{1}{N-1}\;\sum_{i=1}^{N}\;{\left({\vec{v}}_{i}\;-\;\frac{1}{N}\;\sum_{j=1}^{N}\;{\vec{v}}_{j}\right)}^{2}\right]} \end{array}$$ (2)

in order to minimize the effects of bias and noise on time-lapse recorded positions which otherwise leads to a negative *c*_*v*_ at small *τ* values.

The turn angle distribution *p*(*α*) was defined as the fraction of trajectory steps for which their instantaneous turn angle *α*_*i*_ lies between *k*Δ*α* and (*k* + 1)Δ*α* relative to their previous steps. The turn angle is *α*_*i*_ = *θ*_*i*_ − *θ*_*i*−1_, where *θ*_*i*_ is the orientation of the displacement *i*, $\Delta \vec{r_{i}}\equiv {\vec{r}}_{i}-{\vec{r}}_{i-1}$, relative to the horizontal axis. A histogram for the turn angles with a bin size Δ*α* = (*α*_*max*_ − *α*_*min*_)/50 were built. As in reference [21], *n* = 50 was empirically chosen as the number of bins, each one containing a significant sample of data points.

We analyzed if the cellular migration exhibits alternating modes (directional and re-orientation phases). Here, we adopted the following mode definition [13, 21]. From the turn angles *α*(*t*_*i*_) of an individual cell trajectory and a chosen threshold value α*, a directional “flight” starts at a time point *t*_*i*_ if at least *n* > 1 successive steps have *α*(*t*_*j*_) < α*, *j* = *i* + 1, …, *i* + *n*. In turn, a re-orientation flight begins at *t*_*i*_ if *n* > 1 successive time steps have *α*(*t*_*j*_) ≥ α*. The values α* = 15°, 30° and 45° were chosen. Once the migration modes have been defined, their distributions of flight lengths were determined as the fraction of flights with contour lengths *l*. The contour length *l* of a flight starting at a time point *t*_*i*_ is defined as the total distance it traverses. Therefore, $l=\sum_{j=1}^{n}\;\left|{\vec{r}}_{i+j}-{\vec{r}}_{i+j-1}\right|$, where *n* is the number of steps comprising the flight.

In searching for possible coordinated motions, the cell velocity fields in large-field images were used to calculate the two-dimensional orientational order parameter, the spatial pair and velocity correlation functions. The orientational order parameter is defined as

$$\begin{array}{c} \xi =|\frac{1}{N}\;\sum_{i}\;{\hat{v}}_{i}|, \end{array}$$ (3)

where ${\hat{v}}_{i}$ is the unit velocity vector of the cell *i* and 〈…〉 represents the average over all cells. In turn, the spatial pair correlation function is expressed as

$$\begin{array}{c} g\left(x,y\right)=\frac{1}{\rho }\;{\left\langle \sum_{j\neq i}\;\delta (|x\hat{x}+y\hat{y}-\left({\vec{r}}_{i}-{\vec{r}}_{j}\right)\left|\right)\right\rangle }_{i}, \end{array}$$ (4)

where *δ* is the Dirac function, *ρ* = *N*/*A* is the area cell density, and 〈…〉_*i*_ is the average over all reference cells *i*. Finally, the spatial velocity correlation function is given by

$$\begin{array}{c} c_{v}\left(\vec{r}\right)=\frac{{\left\langle \left({\hat{v}}_{i}\cdot {\hat{v}}_{j}\right)\delta \left[x\hat{x}+y\hat{y}-\left({\vec{r}}_{i}-{\vec{r}}_{j}\right)\right]\right\rangle }_{ij}}{{\left\langle \delta \left[x\hat{x}+y\hat{y}-\left({\vec{r}}_{i}-{\vec{r}}_{j}\right)\right]\right\rangle }_{ij}} \end{array}$$ (5)

where $\vec{r}$ is the vector of coordinates (*x*, *y*), and 〈…〉_*ij*_ is the average over all possible cell pairs.

## Results

### Individual cell migration

**Fig 1:**
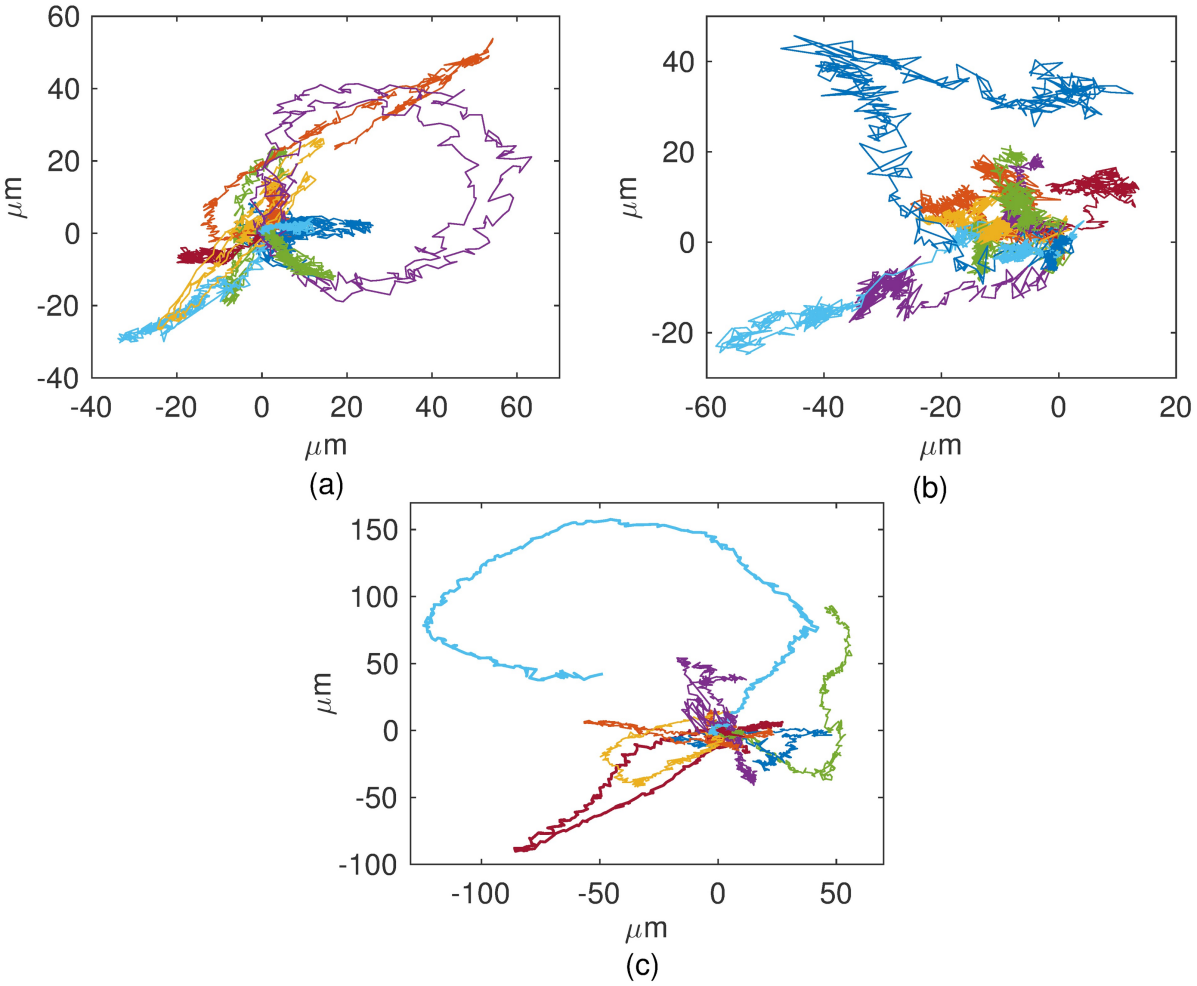
Typical migration tracks of B16F10. Cells on 2D plastic substrates plated at (a) 20, (b) 2000, and (c) 10000 cells per cm^2^. The trajectories were produced by time-lapse recording of cells every 1 min and plotted from the origin.

**Fig 2:**
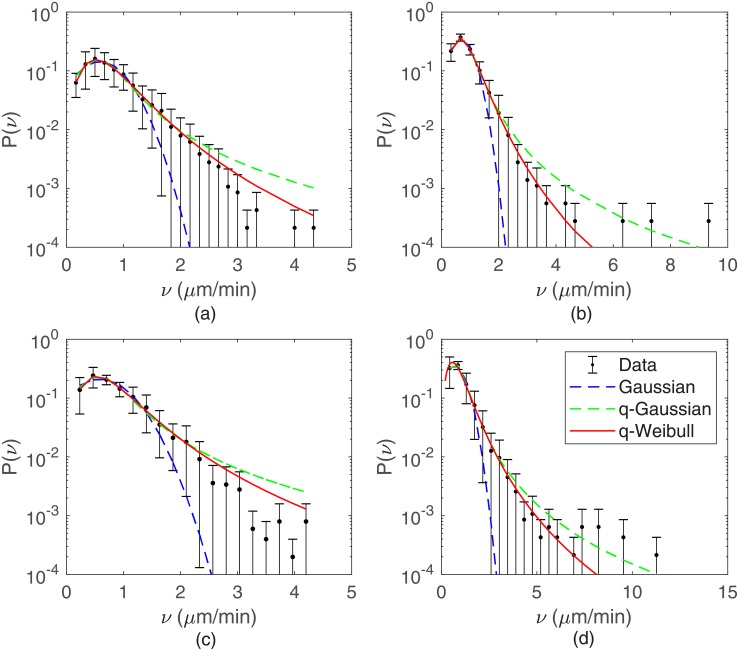
Ensemble speed distributions for B16F10. Cells plated at densities of (a) 20, (b) 100, (c) 2000, and (c) 10000 cells per cm^2^. The solid curves are q-Weibull fits to data. For comparison, Gaussian and q-Gaussian distributions fitted to data are shown (dashed curves). The velocities for every individual cell were merged to form single large data sets. Other values than *k*_*max*_ = 30 were used to fix the speed bin size, generating very similar speed distributions (see S1 PDF).

In Fig 1 typical trajectories for B16F10 cells cultured on monolayers plated at distinct densities are shown. From them speed distributions were determined. As illustrated in Fig 2, the speeds of B16F10 melanocytes are best fitted by q-Weibull distributions [22] expressed by the formula

$$\begin{array}{c} P_{qw}\left(x\right)=p_{0}\frac{rx^{r-1}}{x_{0}^{r}}\exp_{q}\;[-\;{\left(\frac{x}{x_{0}}\right)}^{r}], \end{array}$$ (6)

where exp_*q*_(−*x*) ≡ [1 − (1 − *q*)*x*]^1/(1 − *q*)^ is the q-exponential [23]. Note that for *r* = 1 and *q* ≠ 1 the q-exponential is recovered, whereas in the limit *q* → 1 with *r* ≠ 1 or *r* = 1 the Weibull or the exponential distributions, respectively, are obtained [22]. The values of *q* and *r* obtained for B16F10 cells are listed in Table 1.

**Table 1:** Table with some values obtained by adjustments.

B16F10 density	*q*	*r*	*s*	*κ*	*γ*_*e*_	*q*_*c*_*v*__
20 cells/cm^2^	1.42 ± 0.08	2.38 ± 0.10	1.01 ± 0.30	1.52 ± 0.90	1.50 ± 0.03	2.62 ± 0.30
100 cells/cm^2^	1.37 ± 0.02	2.77 ± 0.02	1.27 ± 0.50	0.43 ± 0.40	1.44 ± 0.02	2.11 ± 0.03
2000 cells/cm^2^	1.40 ± 0.09	2.28 ± 0.20	1.14 ± 0.20	1.68 ± 0.60	1.21 ± 0.02	2.15 ± 0.20
10000 cells/cm^2^	1.37 ± 0.03	2.20 ± 0.08	1.16 ± 0.20	2.46 ± 0.80	1.66 ± 0.03	2.34 ± 0.30

Parameters *q* and *r* characterizing the q-Weibull cell speed distributions, their skewnesses *s* and kurtoses *κ*, and exponents *γ*_*e*_, the slopes of the empirical power-law fittings to average 〈*r*^2^〉 curves. Also, the *q*_*c*_*v*__ values used for fitting velocity autocorrelations *c*_*v*_ are listed.

Independently of fitting protocols, the skewness *s* and kurtosis *κ* of the speed distributions can be calculated from the raw data for cell speeds. These quantities, defined as

$$\begin{array}{c} s=\frac{\mu_{3}}{\mu_{2}^{3/2}}\;\text{and}\;\kappa =\frac{\mu_{4}}{\mu_{2}^{2}}-3, \end{array}$$ (7)

where *μ*_2_, *μ*_3_, and *μ*_4_ are, respectively, the second, third, and fourth moments of the distributions, provide relevant information to guide curve fitting. For B16F10 cells, the values obtained for *s* and *κ* are listed in Table 1. As one can see, these values correspond to asymmetric and leptocurtic speed distributions, hence excluding the Gaussian and q-Gaussian distributions, both symmetric. Consequently, the present analysis points out to a correction in our previous result, contained in reference [21], according which normal (Melan A) and tumoral (B16F10) melanocytes migrate in monolayer culture exhibiting q-Gaussian speed distributions.

**Fig 3:**
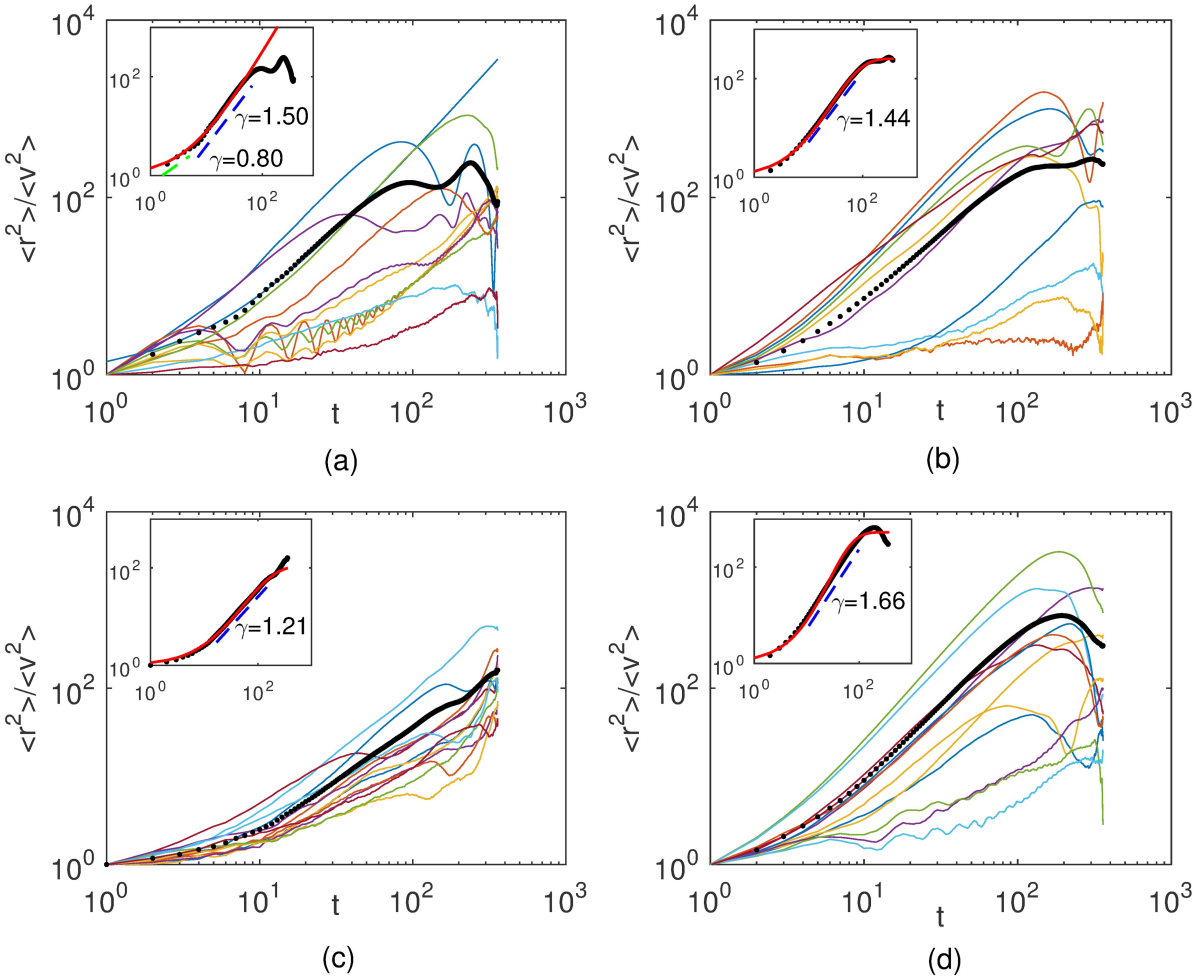
Mean-squared displacements 〈*r*^2^〉 as functions of time for B16F10. Cells plated at (a) 20, (b) 100, (c) 2000, and (c) 10000 cells per cm^2^. The color curves correspond to distinct cell trajectories. The thick black curve is the average of all trajectories. The dashed lines are power law fits to data whose slopes provide the exponents *γ* characterizing the migratory regimes. Mean-squared displacements were divided by 〈*v*^2^〉 in order to put in the same scale cells with very distinct motilities. Insets: Average mean-squared displacements fitted by Eq (9). A crossover in time from a normal to a superdifusive regime is indicated.

The anomalous character of B16F10 migration is confirmed by the scaling in time of the mean-squared displacement given by

$$\begin{array}{c} \left\langle r^{2}\right\rangle ∼t^{\gamma } \end{array}$$ (8)

with *γ* ≠ 1. In Fig 3 are shown log-log plots of mean-squared displacements for distinct individual cells as functions of time. Power-law fittings to empirically chosen linear portions of the average 〈*r*^2^〉 curves were drawn (dashed lines) and their slopes *γ*_*e*_ indicated. In the insets, we have fitted the whole set of experimental data for the average mean-squared displacements by using a nonextensive statistics scenario in which a crossover between two different power-laws is assumed [21, 24]. Specifically, if *x* ≡ 〈*r*^2^〉 satisfy the equation

$$\frac{dx}{dt}=\mu_{1}x+\left(\mu_{2}-\mu_{1}\right)x^{\lambda },$$

*μ*_1_ < *μ*_2_, its solution

$$\begin{array}{c} x={\left[1-\frac{\mu_{2}}{\mu_{1}}+\frac{\mu_{2}}{\mu_{1}}e^{\left(1-\lambda \right)\mu_{1}t}\right]}^{\frac{1}{1-\lambda }} \end{array}$$ (9)

provides the parameters *μ*_1_, *μ*_2_, and λ for fitting the experimental data. The red curves shown in the insets correspond to such fits and their parameter values are listed in Table 1. Its worth to notice that the nonextensive fittings become worse at the lowest densities tested. Indeed, for 20 cells/cm^2^, a sharp change from one to another power-law is observed

**Fig 4:**
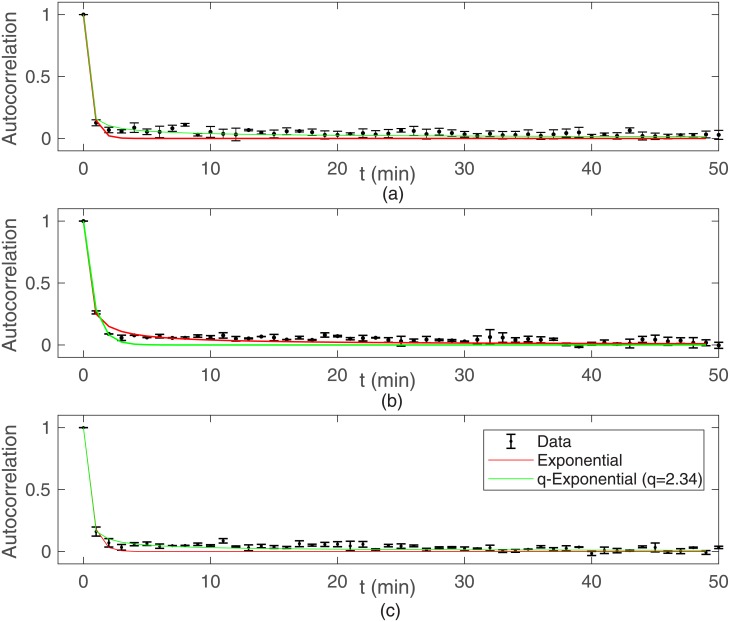
Velocity autocorrelation functions for B16F10. Cells plated at (a) 20, (b) 2000, and (b) 10000 cells per cm^2^. Velocities were defined as (displacement vector)/(time-lapse) for each cell track.

In Fig 4 are shown our experimental results for the velocity autocorrelation functions determined through Eq (2). B16F10 cells exhibit velocity correlations decaying q-exponentially in time with very short characteristic time scales, typically ∼ 0.5 − 0.7 min, for all densities plated. The *q*_*c*_*v*__ values obtained by fitting velocity autocorrelation *c*_*v*_ with q-exponentials are listed in Table 1.

**Fig 5:**
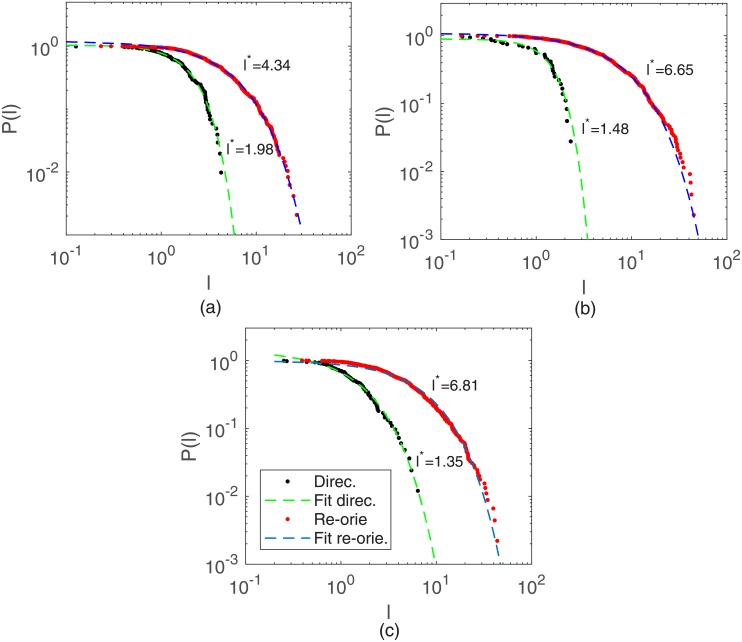
Cumulative distributions of flight lengths for B16F10. Cells plated at (a) 20, (b) 2000, and (c) 10000 cells per cm^2^. A threshold α* = 30° was used. The continuous curves correspond to exponential fittings to the data with characteristic contour lengths l*.

As done in reference [21], every individual cell track was subdivided into directional and reorientation “flights”. The former (latter) are comprised of successive displacements whose turn angles are always smaller (greater) than a fixed threshold [13]. Our results, illustrated in Fig 5, reveal that both flight types are exponentially distributed for turn angle thresholds α* ranging from 15° − 45°. The directional flights have smaller characteristic number of steps than reorientation flights and, consequently, shorter characteristic contour lengths l*.

**Fig 6:**
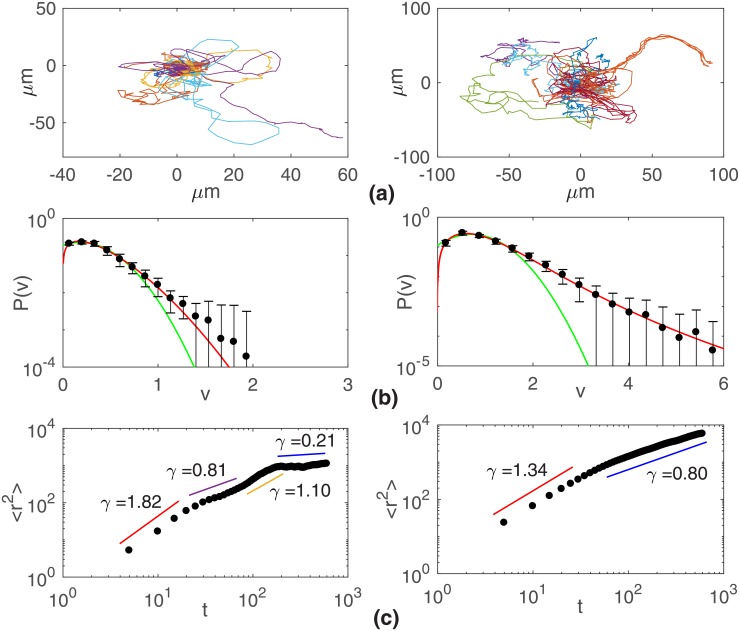
Migratory traits of MDCK. Cells plated at 1190 (left) and 11,900 (right) cells per cm^2^. (a) Typical cell tracks, (b) speed distributions, and (c) mean-squared displacements 〈*r*^2^〉. As for B16F10 cells, q-Weibull speed distributions and anomalous diffusive motion are observed.

Similar results were found for the migration of MDCK cells, as shown in Fig 6. Since these cells exhibit a greater motility than B16F10 cells, they were tested for the emergence of collective migration. Nevertheless, of special relevance is the complex behavior of the mean squared displacement exhibited by MDCK cells (see Fig 6(c)). At low density, sub- and superdiffusive regimes alternate in time, a trait not observed in B16F10 cells.

### Searching for collective migration

**Fig 7:**
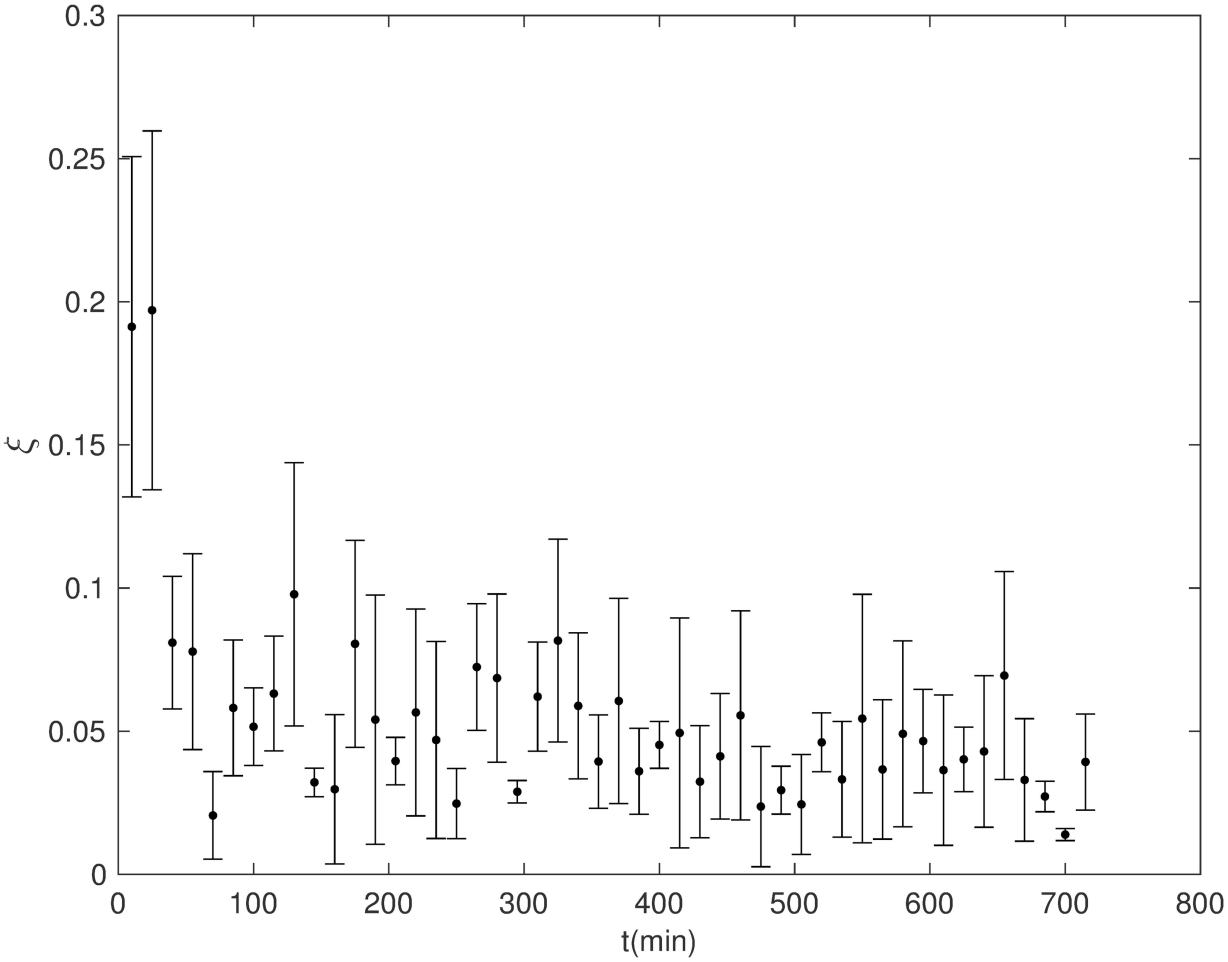
Evolution in time of the order parameter *ξ* for MDCK. Cells plated at 11,900 cells per cm^2^. The small *ξ* values indicate the absence of a long-range ordered cell migration.

Fig 7 illustrates the evolution in time of the order parameter *ξ*, defined in Eq (3). At the two densities tested, the emergence of a coherent dynamics of MDCK cells characterized by a long-range alignment of their velocities seems to be elusive. At most, the very small, but non vanishing values of *ξ* can suggest that the system is bordering the transition point. However, the existence of short-ranged, high-ordered moving clusters is still possible.

**Fig 8:**
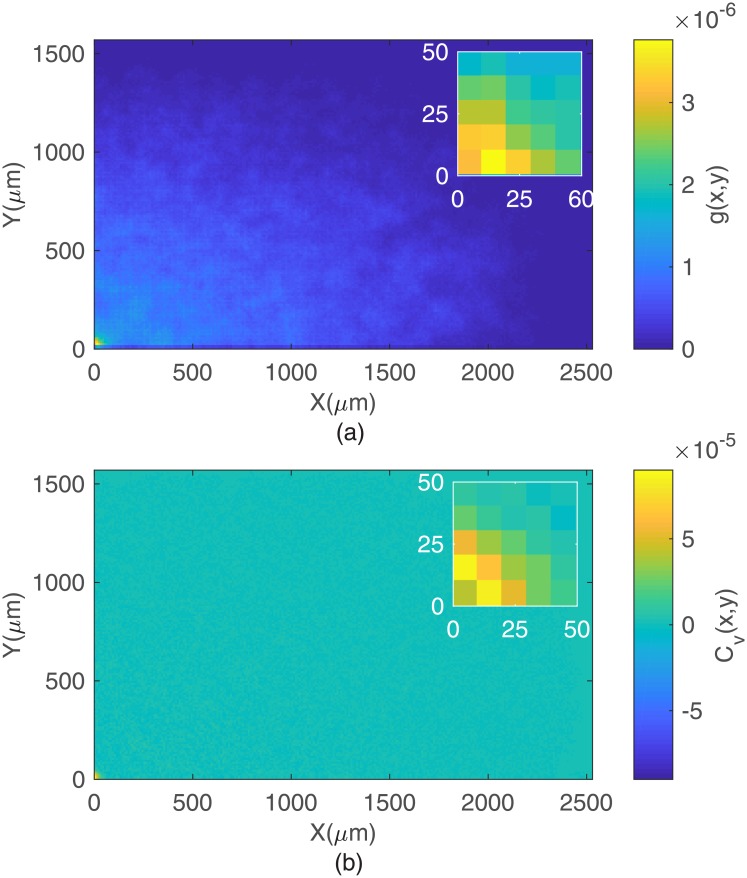
Pair and spatial velocity correlation to MDCKS cells. (a) Pair correlation *g*(*x*, *y*) and (b) spatial velocity correlation functions for MDCK cells plated at 11,900 cells per cm^2^, the larger density tested. Significant spatial correlations associated to the formation of small cell clusters are observed only at short distances, as shown in the inset.

In the quest for local correlations among individual cell motions, both the pair *g*(*x*, *y*), defined in Eq (4), and the spatial velocity correlation functions, defined in Eq (5), respectively, were computed. The pair correlation function g(x,y) quantifies the probability per unit area (normalized by the area density *ρ*) of finding another cell at the location $\vec{x}=\left(x,y\right)$ away from the reference cell. The results for *g*(*x*, *y*) are shown in Fig 8(a). Larger correlations are observed only at short distances *l* ∼ 14 *μ*m, roughly the diameter of a single cell, and at the higher density tested. Thus, MDCK cells can form small clusters in which a cell is positioned very close to a nearest neighbor. Cluster size distribution functions decaying as stretched exponentials with small characteristic sizes (of the order of 1.12 cells) provide further support to those findings (data not shown). Additionally, the spatial velocity correlation function *c*_*v*_(*x*, *y*) quantifies the similarity between the unit velocities (measured by their scalar product) of two cells separated by the vector $\vec{x}=\left(x,y\right)$ at the time *t*. The results for *c*_*v*_(*x*, *y*) are shown in Fig 8(b). For the two densities, there is evidence of significant correlation in velocity between close neighbor cells. This observation is consistent with the possibility, raised by *g*(*x*, *y*), of small dynamical clusters to form, within which cell motions are significantly correlated.

### Simulational tests

Concerning the origin of the q-Weibull distributions for cell speeds, we focused on a major trait of cell migration: persistence. Its effect on minimal models of single and collective cell motion was studied through computer simulations. Specifically, two models were considered, namely, a correlated random walk and the Vicsek model [25]. In both cases, *N* particles are randomly dispersed in a two-dimensional plane substrate and move off-lattice with a constant speed *v*_0_. The initial direction of motion, specified by the polar angle *θ*_*i*_, *i* = 1, 2, …, *N*, is chosen at random. After each time step Δ*t*, every particle changes its direction of motion according to the rule

$$\begin{array}{c} \theta_{i}\left(t+\Delta t\right)=\phi_{i}\left(t+\Delta t\right)+\eta , \end{array}$$ (10)

where *η* ∈ [−*ξ*, +*ξ*] is a white noise with the intensity *ξ* ranging in the interval [0, *π*]. For the correlated random walk model, *ϕ*_*i*_(*t* + Δ*t*) = *θ*_*i*_(*t*), i. e., the polar angle specifying the direction of motion of the particle *i* at the previous time. In turn, for the Vicsek model,

$$\begin{array}{c} \phi_{i}\left(t+\Delta t\right)=Arg\;[\sum_{j|r_{ij}\leq r_{0}}\;\hat{s_{j}}\left(t\right)], \end{array}$$ (11)

where the function *Arg* returns the polar angle specifying the direction of the average vector $\sum_{j}\;{\hat{s}}_{j}$, in which ${\hat{s}}_{j}$ is the unit vector oriented along the direction of motion of the particle *j*, and $r_{ij}=\left|{\vec{r}}_{i}-{\vec{r}}_{j}\right|$. Thus *ϕ*_*i*_ corresponds to an orientation parallel to the mean velocity of the neighboring particles (including *i*) located within a radius *r*_0_ around *i*. So, the evolution in time of particle positions is described by the map

$$\begin{array}{c} {\vec{r}}_{i}\left(t+\Delta t\right)={\vec{r}}_{i}\left(t\right)+v_{0}\Delta t\;{\hat{e}}_{i}\left[\phi_{i}\left(t+\Delta t\right)\right], \end{array}$$ (12)

with the discrete time step Δ*t* ≡ 1.

Since in both models all particles move with a constant speed, the associated speed distribution is a Dirac *δ*-function. We refer to it as the “microscopic” speed distribution. However, the sampling time interval in which the particle positions are recorded in an experiment is usually much larger than the microscopic time interval Δ*t*. Indeed, the experimentally measured velocity is ${\vec{v}}_{i}\left(t\right)=\left[{\vec{r}}_{i}\left(t\right)-{\vec{r}}_{i}\left(t-\tau \right)\right]/\tau$, where *τ* is the sampling time interval. A speed distribution *p*(*v*), termed as “macroscopic”, corresponds to such velocities.

**Fig 9:**
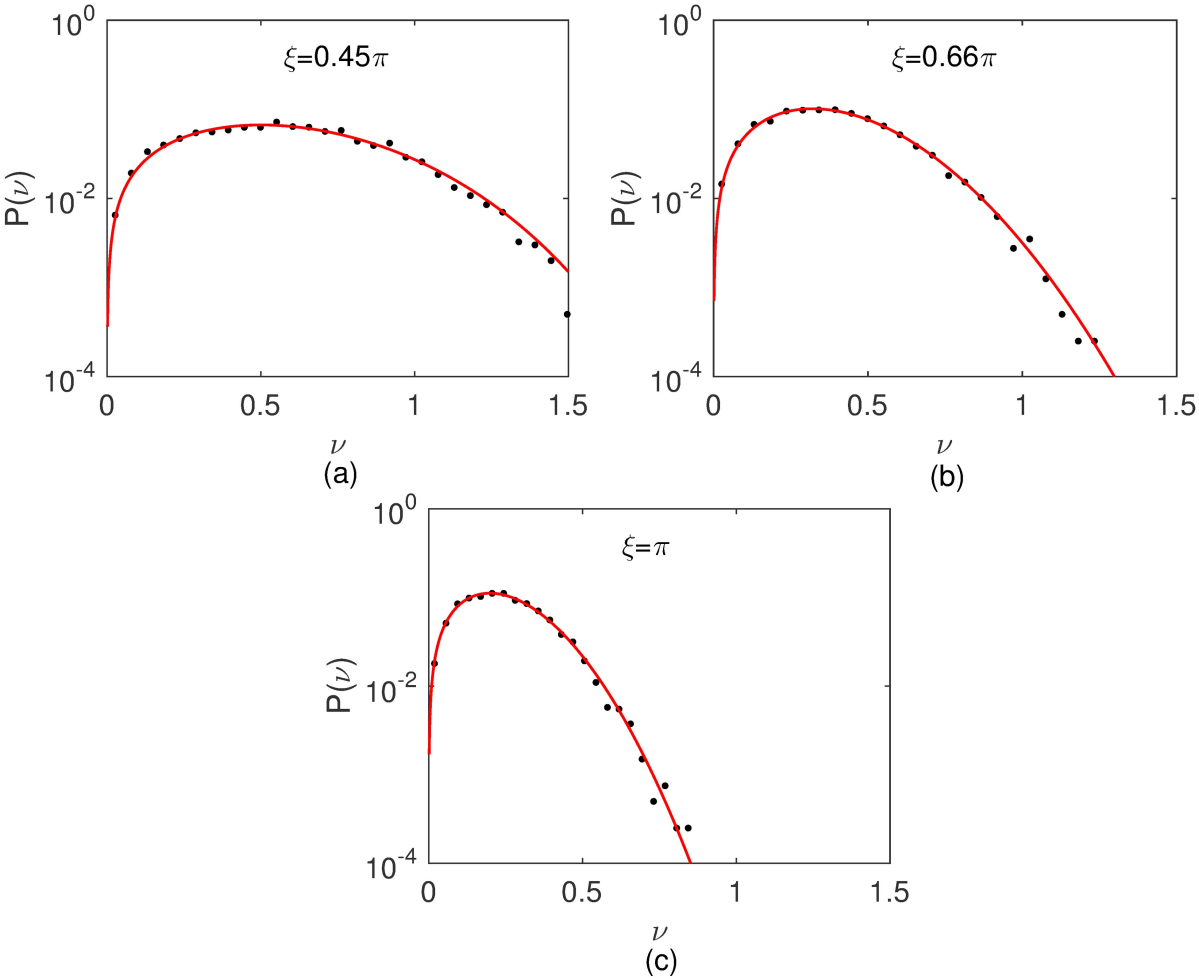
Macroscopic speed distributions for the correlated random walk model at three different noise levels. The sampling time is fixed in *τ* = 50. The solid curves in the histograms are q-Weibull fits to data.

**Fig 10:**
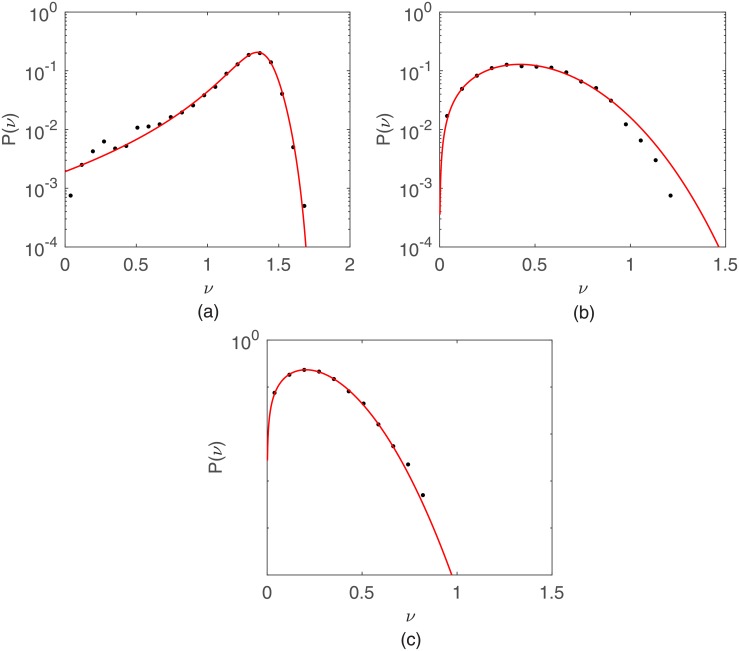
Macroscopic speed distributions for the Vicsek model. The same as in Fig 9 but for the Vicsek model.

In Figs 9 and 10 are shown the simulated macroscopic speed distributions for, respectively, the correlated random walk and Vicsek models. The value *τ* = 50 was fixed and three noise intensities *ξ* = 0.45*π*, 0.66*π*, and *π* were tested. For both models the stationary speed distributions are q-Weibull in the presence of noise (*ξ* ≠ 0), but a Dirac *δ*-function in the absence of noise (*ξ* = 0). In particular, at the random walk limit (*ξ* = *π*), the speed distributions converge to Weibull functions with *q* → 1 and *r* = 2, i. e., 2*D* Maxwellians.

## Discussion

The motility of epithelial cell lines MDCK (non-tumorigenic) and B16F10 (tumoral) in the absence of external chemotactic signals (random conditions) was investigated through time-lapse microscopy. As can be seen in Figs 1, 3 and 6(a) and 6(c) even within a specified cell line, the cells exhibit very diverse migratory behaviors, from hardly motile to persistently migrating cells along a chosen direction or, yet, cells that either oscillate back and forth or turn around their start positions. Granted that individual cells may have higher or lower motility capabilities, these cells were grouped into two phenotypes based on their mean-squared displacement behaviors. An analysis of such phenotypes was performed in the Supporting information(S1 PDF).

Regardless this diversity of individual cell behavior, a well defined average motility pattern emerges: these epithelial cells migrate either subdiffusively or normally at short time scales, but performs a superdiffusion at large time scales. Furthermore, the speed distributions for both MDCK and B16F10 cells are q-Weibull and the cell velocities autocorrelations are q-exponentials with very short characteristic correlation times. Concerning the non-Gaussian migration of these cells, three main results deserve special attention.

Firstly, two average regimes associated to directed motion with angular fluctuations are dominant for B16F10 cells even at the low densities tested. Single B16F10 cells migrating under external chemotactic gradients or experimenting the signalling from multiple cell clusters randomly dispersed on the culture plate [21], perform a normal or subdiffusive migration (〈*r*^2^〉 ∼ *t*^*γ*^, with *γ* ≤ 1) at short and a superdiffusive motion (〈*r*^2^〉 ∼ *t*^*γ*^, with *γ* > 1) at long time scales. However, for MDCK cells plated at the lower density of 1190 cells per cm^2^, the mean-squared displacement exhibit, as shown in Fig 6(c), a more complex behavior. Instead of only two regimes, a complex transient characterized by successive alternating regimes of motion occurs. Four regimes separated by three crossovers are observed. The first and the third ones are superdiffusive, whereas the second and the fourth are subdiffusive. In particular, the first regime is very close to a ballistic motion, a sign of persistent random walks, while the fourth one, strongly subdiffusive, is possibly due to the circulation of cells within constrained regions in the absence of significant chemotactic gradients. (See Fig 6(a)) A similar behavior is exhibited by less motile B16F10 subpopulations at the lower density tested. Since our previous [21] and present results for turn angle (see S1 PDF), speed, and flight length distributions reveal that both directions and speeds of cell migration fluctuate, we are facing a scenario consistent with the theoretical case considered by Peruani and Morelli [26].

These authors demonstrated that the interplay of independent speed and angular fluctuations gives rise to a sequence of alternating regimes of motion for two distinct angular dynamics correspondent to persistent and directed random walks. Moreover, they showed that such complex transients are not observed when speed fluctuations are absent. Therefore, our results are an experimental evidence of complex transients similar to those predicted by Peruani and Morelli [26]. If only one regime is present at intermediate time scales, the resulting sequence of three diffusive behaviors for MDCK cell migration is yet consistent with a persistent random walk highly plausible for cell motility [26]. In this walk, a particle starts moving along a randomly chosen direction and diffusively explores other directions, therefore demanding a characteristic time to be found pointing with equal probability to any direction. In addition, the interplay of speed and angular fluctuations can also give rise to the simpler average behavior of B16F10 cells, characterized by a single crossover in 〈*r*^2^〉, if for these cells the ratio between speed and angular change rates is very small. It is worth to mention that for animal cells in culture is very possible that speed and angular fluctuations are correlated, therefore altering the aforementioned sketch.

Secondly, the speeds of MDCK and B16F10 cells are q-Weibull distributed. Furthermore, as the density of plated cells increases, the parameters *q* and *r* characterizing the q-Weibull functions vary. Our experimental data and model simulations indicate that *q* → 1 and *r* → 2, leading to the speed distribution *p*(*v*) ∼ *v* exp(−*kv*^2^) (*k* a constant). This Weibull distribution is the two-dimensional version of the Maxwellian *p*(*v*) ∼ *v*^2^ exp(−*kv*^2^) (*k* = *m*/2*k*_*B*_*T*) valid not only for a three-dimensional classical ideal gas, but also for any system in which quantum effects are negligible, independently on the correlations introduced by the conservative interactions between their particles [27]. In the culture of animal cells, non-conservative interactions are probably in action, but conservative forces between individual cells can usually be neglected. Since, according our results, the cell speed distributions approach a 2*D* Maxwellian, the relevance of these non-conservative interactions decreases as the density of cells increases. Hence, migrating cells tend to perform Brownian-like motions at asymptotically large cell densities. Further support to this scenario is provided by our findings that, as the cell density rises, the characteristic lengths of directional flights decrease whereas those associated to reorientation flights increase.

Regarding cell speed distributions, our present findings improves and corrects our previous published results [21]. The following facts justify this correction: (i) more extensive experimental data, particularly for MDCK cells, led to a better statistics mainly at the small range of cell speeds, where a q-Gaussian significantly deviates from the measured data. (ii) A statistical analysis of the whole raw data on cell speeds indicates that the speed distribution is asymmetric, therefore ruling out the symmetric q-Gaussian as an adequate fitting. We emphasize that this statistical analysis based on the evaluation of the moments of a distribution is absolutely independent on data fitting protocols. (iii) It is the q-Weibull distribution (asymmetricas demanded by the skewness of the experimental data) that has a 2D Maxwellian distribution at the limit of a Brownian cell migration. This asymptotic behavior is, for the physicists, a specially relevant feature that gives further support to the consistency of the measured data. Therefore, correcting our previous claim that the cell speed distributions are q-Gaussians is a major result of the present manuscript.

Additionally, MDCK and B16F10 cells do not exhibit long-range spatio-temporal velocity correlations. When randomly dispersed and crawling onto a 2*D* rigid substrate without external chemotactic sources or mechanical guidance, these epithelial cells can not self-organize themselves into ordered collective motions. In contrast, as observed by Angelini *et al*. [28], onto soft substrates MDCK cells can migrate collectively in response to cooperative cell-driven patterns of substrate deformation. The collective migration of MDCK cells is also triggered by just releasing a free surface in a confluent monolayer [29, 30]. The epithelial monolayer’s motility involves both complex and coordinated long-range motions within the epithelium, and a fingering of the borders elicited by very active “leader cells” that precede a small cohort. It seems that, in response to the free surface, a strong mechanical tension and signalling between the leaders and their followers mediates the collective migration.

Thirdly, our simulations demonstrate that persistence is the simplest yet sufficient ingredient to generate q-Weibull distributions for cell speeds. Contrasting to our previously reported results [21], no correlated random walk characterized by complex and “ad hoc” step length distributions, such as q-Gaussians or other non-extensive functions, is necessary to generate non-Gaussian speed distributions. Indeed, non-interacting particles moving with a constant speed along directions that changes continuously at random within a specified angular dispersion generate q-Weibull speed distributions at “macroscopic” sampling times. The stronger is the persistence, i. e., smaller is the turn angle fluctuation range, more the q-Weibull deviates from the 2*D* Maxwellian (*q* → 1 and *r* = 2) limit associated to a unbiased random walk with a turn angle fluctuation range of 2*π*. Alignment interactions between particles can elicit or reinforce persistent, collective motions and, therefore, generate q-Weibull speed distributions. Our simulational results for the Vicsek model confirm this. In living active matter, interactions such as steric exclusion in suspensions of bacteria [31], hydrodynamic coupling in dense bacterial colonies [32, 33], or mechanical deformations in a soft substrate where cells migrate [28] are also candidates to produce q-Weibull speed distributions. However, speed fluctuations alone arising, for instance, from the intrinsic stochasticity of the cellular engine driving individual motion do not lead to q-Weibull distributions unless they are distributed in that way.

## Conclusions

The migration of epithelial cells MDCK (normal) and B16F10 (tumoral) onto 2*D* rigid substrates and under random conditions were characterized through time-lapse microscopy. The major findings are that these cell lines exhibit anomalous diffusion with q-Weibull speed distributions and short-ranged persistent motions comprised by directional flights with contour lengths exponentially distributed. This persistence is a sufficient feature to generate q-Weibull speed distributions, as our simulations demonstrate. Also, fluctuations in cell speeds are responsible for a complex behavior of the mean-squared deviation which can exhibits alternating sub- and superdiffusive regimes at low densities of plated cells. Finally, it seems that these epithelial cells do not self-organize into collective motions when plated dispersedly on a rigid plastic substrate and in the absence of any extrinsic chemotactic gradient and mechanical cues. At the cell densities tested, which are far from confluence, spatial pair and speed correlations indicate only the formation of small clusters involving close neighbor cells. This result may have relevance for tissue engineering and the design of materials to interact with cells because the properly colonization of rigid prosthetic devices or inorganic interfaces by cells through self-organization is maybe hardly constrained.

## Supporting information

S1 PDF Extra results and explications. In the PDF we can check the extra results concerning: bin sizes and speed distributions, mean squared displacements for distinct migratory phenotypes and turn angle distributions.
(PDF)

S1 File Data from the B16F10 cells trajectories. Individual trajectories of each cell over time for density of 20 *cel*/*cm*^2^. (available in http://www.posfisicaaplicada.ufv.br/?pageid=2714).
(ZIP)

S2 File Data from the B16F10 cells trajectories. Individual trajectories of each cell over time for density of 100 *cel*/*cm*^2^. (available in http://www.posfisicaaplicada.ufv.br/?pageid=2714).
(ZIP)

S3 File Data from the B16F10 cells trajectories. Individual trajectories of each cell over time for density of 2000 *cel*/*cm*^2^. (available in http://www.posfisicaaplicada.ufv.br/?pageid=2714).
(ZIP)

S4 File Data from the B16F10 cells trajectories. Individual trajectories of each cell over time for density of 1000 *cel*/*cm*^2^. (available in http://www.posfisicaaplicada.ufv.br/?pageid=2714).
(ZIP)

S5 File Data from the MDCK cells trajectories. Individual trajectories of each cell over time for density of 1190 *cel*/*cm*^2^. (available in http://www.posfisicaaplicada.ufv.br/?pageid=2714).
(ZIP)

S6 File Data from the MDCK trajectories. Individual trajectories of each cell over time for density of 11900 *cel*/*cm*^2^. (available in http://www.posfisicaaplicada.ufv.br/?pageid=2714).
(ZIP)

S1 Video Film of MDCK. This film was obtained with superposition of all the pictures in all the time. (available in http://www.posfisicaaplicada.ufv.br/?pageid=2714).
(WMV)

S2 Video Film of Viscseck model. This film was obtained used the position of particles in a simulation whith Vicsek Model and r = 2.80rad in all time. (available in http://www.posfisicaaplicada.ufv.br/?pageid=2714).
(WMV)

## References

[1] Bray D (2001) Cell movements: from molecules to motility. New York: Garling Publishing.

[2] Thiery JP, Acloque H, Huang RYJ, Angela Nieto M (2009) Epithelial-mesenchymal transitions in development and disease. Cell 139: 871–890. 10.1016/j.cell.2009.11.007 19945376

[3] Carnell MJ, Insall RH (2011) Actin on disease—Studying the pathobiology of cell motility using *Dictyostelium discoideum*. Semin. Cell Develop. Biol. 22: 82–88. 10.1016/j.semcdb.2010.12.003 21145982

[4] Blitterswijk CV et al eds. (2008) Tissue engineering. London: Academic Press

[5] Marchetti MC, Joanny JF, Ramaswamy S, Liverpool TB, Prost J, Rao M. et al (2013) Hydrodynamicsof soft active matter. Rev. Mod. Phys. 25: 1143–1189.

[6] Ramaswamy S (2010) The mechanics and statistics of active matter. Ann. Rev. Condens. Matter Phys. 1: 323–345. 10.1146/annurev-conmatphys-070909-104101

[7] Wiswanathan GM, da Luz MGE, Raposo EP, Stanley HE (2011) The Physics of Foraging (Cambridge University Press, Cambridge).

[8] Parsons JT, Horwitz AR, Schwartz MA (2010) Cell adhesion: integrating cytoskeletal dynamics and cellular tension. Nature Rev. Mol. Cell Biol. 11: 633–643. 10.1038/nrm2957 20729930 PMC2992881

[9] Codling EA, Plank MJ, Benhamou S (2013) Random Walk models in biology. J. R. Soc. Interface 5: 813–834. 10.1098/rsif.2008.0014 PMC2504494 18426776

[10] Li L, Cox EC, Flyvberg H (2011) ‘Dicty dynamics’: *Dictyostelium* motility as persistent random motion. Phys. Biol 8: 046006 10.1088/1478-3975/8/4/046006 21610290 PMC3148805

[11] Li L, Nørrelykke SF, Cox EC (2008) Persistent cell motion in the absence of external signals: a search strategy for eukaryotic cells. PLoS ONE 3(5): e2093 10.1371/journal.pone.0002093 18461173 PMC2358978

[12] Rieu JP, Upadhyaya A, Glazier JA, Ouchi NB, Sawada Y (2000) Diffusion and deformations of single Hydra cells in cellular aggregates. Biophys. J. 79: 1903–1914. 10.1016/S0006-3495(00)76440-X 11023896 PMC1301082

[13] Potdar AA, Jeon J, Weaver AM, Quaranta V, Cummings PT (2010) Human mammary epithelial cells exhibit a bimodal correlated random walk pattern. PLoS One 5(3): e9636 10.1371/journal.pone.0009636 20224792 PMC2835765

[14] Selmeczi D, Mosler S, Hagedorn PH, Larsen NB, Flyvbjerg (2005) Cell motility as persistent random motion: theories from experiments. Biophys. J. 89: 912–931. 10.1529/biophysj.105.061150 15951372 PMC1366641

[15] Updhyaya A, Rieu JP, Glazier JA, Sawada Y (2001) Anomalous diffusion and non-Gaussian velocity distribution of Hydra cells in cellular aggregates. Phys. A 293: 549–558. 10.1016/S0378-4371(01)00009-7

[16] Uhlenbeck GE, Ornstein S (1930) On the theory of Brownian motion. Phys. Rev. 36: 823–841. 10.1103/PhysRev.36.823

[17] Lévy P (1937) Théorie de l’addition des variables aléatoires. Paris: Gauthier-Villars.

[18] Bartumeus F, Peters F, Pueyo S, Marrase C, Catalan J (2003) Helical Lévy walks: adjusting searching statistics to resource availability in microzooplankton. Proc. Natl. Acad. Sci. USA 100: 12771–12775. 10.1073/pnas.2137243100 14566048 PMC240693

[19] Harris TH, Banigan EJ, Christian DA, Konradt C et al (2012) Generalized Lévy walks and the role of chemokines in migration of effector CD8^+^ T cells. Nature 486: 545–548. 10.1038/nature11098 22722867 PMC3387349

[20] Miller MJ, Wei SH, Parker I, Cahalan MD (2002) Two-photon imaging of lymphocyte motility and antigen response in intact lymph node. Science 296: 1869–1873. 10.1126/science.1070051 12016203

[21] da Silva PCA, Rosembach TV, Santos AA, Rocha MS, Martins ML (2014) Normal and Tumoral Melanocytes Exhibit q-Gaussian Random Search Patterns. PLoS ONE 9: e104253 10.1371/journal.pone.0104253 25203532 PMC4159146

[22] Picoli S Jr, Mendes RS, Malacarne LC (2003) q-exponential, Weibull, and q-Weibull distributions: an empirical analysis. Physica A 324: 678–688. 10.1016/S0378-4371(03)00071-2

[23] Tsallis C (2009) Introduction to Nonextensive Statistical Mechanics. New York: Springer.

[24] Tsallis C, Bemski G, Mendes RS (1999) Is re-association in folded proteins a case of nonextensivity? Phys. Lett. A 257: 93–98. 10.1016/S0375-9601(99)00270-4

[25] Vicsek T, Czirók A, Ben-Jacob E, Cohen I, Schochet O (1995) Novel type of phase transition in a system of self-driven particles. Phys. Rev. Lett. 75: 1226–1229. 10.1103/PhysRevLett.75.1226 10060237

[26] Peruani F, Morelli LG (2007) Self-propelled particles with fluctuating speed and direction of motion in two dimensions. Phys. Rev. Lett. 99: 010602 10.1103/PhysRevLett.99.010602 17678144

[27] Le Bellac M, Mortessagne F, Batrouni GG (2010) Equilibrium and Non-Equilibrium Statistical Thermodynamics. Cambridge: Cambridge University Press.

[28] Angelini TE, Hannezo E, Trepat X, Fredberg JJ, Weitz DA (2010) Cell migration driven by cooperative substrate deformation. Phys. Rev. Lett. 104: 168104 10.1103/PhysRevLett.104.168104 20482085 PMC3947506

[29] Poujade M, Grasland-Mongrain E, Hertzog A, Jouanneau J, Chavrier P, Ladoux B, Buguin A, Silberzan P (2007) Collective migration of an epithelial monolayer in response to a model wound. Proc. Natl. Acad. Sci. USA 104: 15988–15993. 10.1073/pnas.0705062104 17905871 PMC2042149

[30] Petitjean L, Reffay M, Grasland-Mongrain E, Poujade M, Ladoux B, Buguin A, Silberzan P (2010) Velocity fields in collectively migrating epithelium. Biophys. J. 98: 1790–1800. 10.1016/j.bpj.2010.01.030 20441742 PMC2862185

[31] Rabani A. Ariel G, Be’er A (2013) Collective motion of spherical bacteria. PLoS ONE 8: e83760 10.1371/journal.pone.0083760 24376741 PMC3869797

[32] Wolgemuth CW (2008) Collective swimming and the dynamics of bacterial turbulence. Biophys. J. 95: 1564–1574. 10.1529/biophysj.107.118257 18469071 PMC2483759

[33] Petroff AP, Wu XL, Libchaber A (2015) Fast-moving bacteria self-organize into active two-dimensional crystals of rotating cells. Phys. Rev. Lett. 114: 158102 10.1103/PhysRevLett.114.158102 25933342

