# Supplementary material for: Anomalous diffusion and q-Weibull velocity distributions in epithelial cell migration
Source: PLoS One. 2017 Jul 10;12(7):e0180777. doi: 10.1371/journal.pone.0180777 (PMC5507264; doi:10.1371/journal.pone.0180777)
Supplement: S1 PDF — In the PDF we can check the extra results concerning: bin sizes and speed distributions, mean squared displacements for distinct migratory phenotypes and turn angle distributions. (PDF) [file pone.0180777.s001.pdf]

Supporting information — Anomalous diffusion and q-Weibull velocity distributions in epithelial cell migration.

[1]**Tatiane S. V. Podestá,** [1]**Tiago V. Rosembach,** [2] **Anésia A. Santos, and** [1,3]**Marcelo L. Martins**
[1]Departamento de Física, [2]Departamento de Bioquímica,
Universidade Federal de Viçosa, 36570-000, Viçosa, Minas Gerais, Brazil
[3] National Institute of Science and Technology for Complex Systems, Brazil

1 Cell trajectories and migration movies

The experimental data can be accessed in http://www.posfisicaaplicada.ufv.br/?page_id=2714. The archives on cell tracking contain the time series for the centroid positions of the observed cells. We also attached movies to illustrate the dynamics of MDCK cell migration in monolayer culture under random motility conditions.

2 Bin sizes and speed distributions

In the manuscript, the distribution $p(\nu)$ was built using a fixed number $n = 30$ empirically chosen aiming to generate the largest number of bins, each one containing statistically significant sample of data points. Here, it is demonstrated that cell speed distributions $p(\nu)$ built using different n values, consequently with distinct bin sizes $\Delta\nu = (\nu_{max} - \nu_{min})/n$, have the same functional forms and similar characteristic parameters. The speed distributions are depicted in Figure 1.

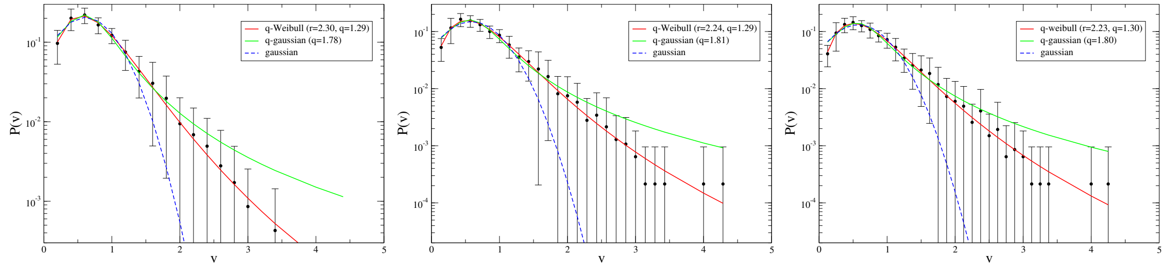

(a) (b) (c)

Figure 1: Experimentally measured ensemble speed distributions for B16F10 cells plated at 20 cells per cm^2 using distinct bin sizes $\Delta\nu$. In (a) $n = 25$, (b) $n = 35$, and (c) $n = 40$. For comparison, q-Weibull, q-Gaussian and Gaussian distributions were fitted to data (color curves). The velocities for every individual cell were merged to form single large data sets.

3 Mean squared displacements for distinct migratory phenotypes

The epithelial cell lines tested exhibit distinct migratory phenotypes, varying from hardly motile to persistently migrating cells or, yet, cells that oscillate around their start positions. Here, we further discuss the diversity of cell motility behaviors.

In Figure 2 are shown the mean-squared displacements (MSD) for distinct B16F10 cells. These cells were grouped into two classes: those having a monotonic and those exhibiting an oscillatory MSD. Monotonic MSDs are associated to more motile cells. Such cells exhibit a crossover from a normal diffusion at short to a superdiffusive migration at long time scales. From these findings we can infer that in more motile cells both the direction of lamellipodium protrusion fluctuates less and the lamellipodia are more stable.

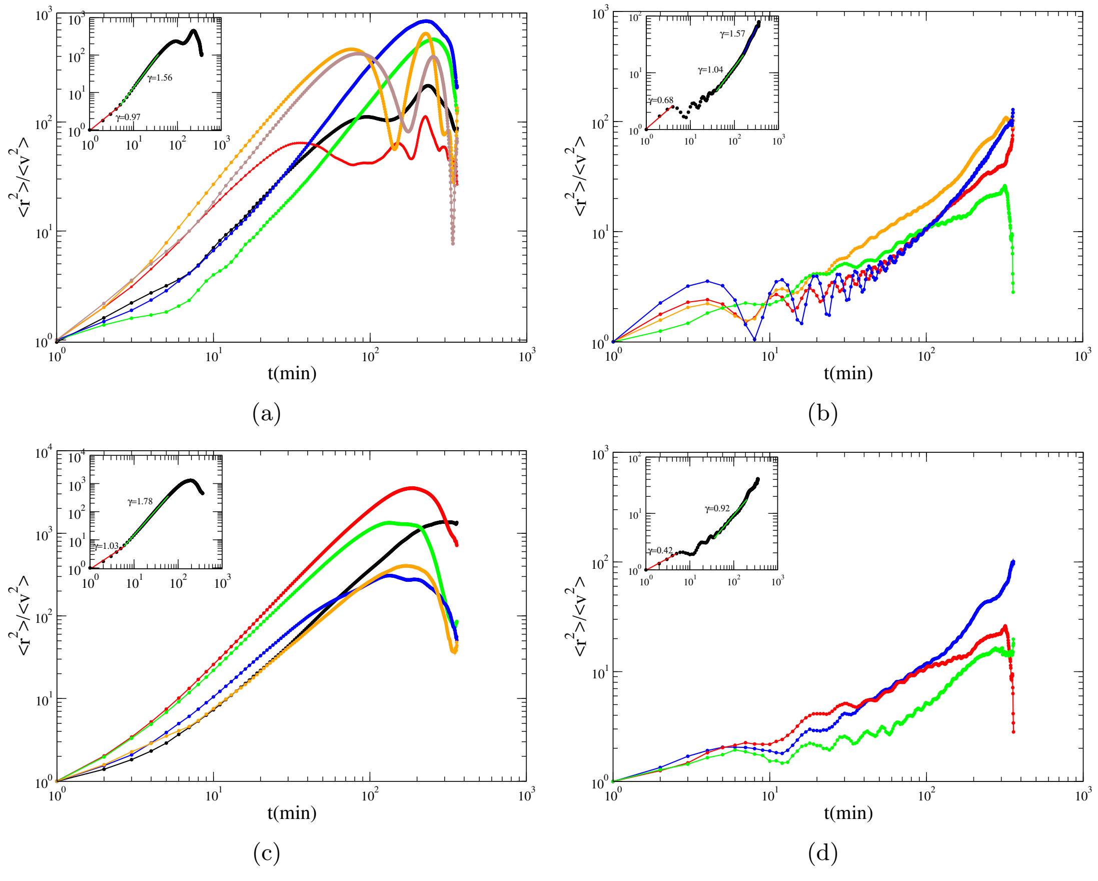

Figure 2: MSD $\langle r^2 \rangle$ as functions of time for B16F10 cells plated at 20 [(a) and (b)] and 10000 [(c) and (d)] cells per cm^2. The color curves correspond to distinct cell trajectories. MSDs were divided by $\langle v^2 \rangle$ in order to put in the same scale cells with very distinct motilities. Insets: Average mean-squared displacements fitted by power laws.

In contrast, oscillatory MSDs characterize less motile cells for which a crossover from subdiffusive at short to normal diffusion at long time scales is observed. Actually, at low density, our results suggest a complex transient behavior for such less motile cells. Instead of a normal diffusion, at intermediate scales the migratory regime is normal, but superdiffusive at long time scales.

Furthermore, it seems that the density of plated cells neatly affects the less motile phenotype. Indeed, there is a strong damping of the MSD oscillations and an increasing homogeneity of cell migration as the density of plated cells rises. The complex transient behavior of such cells is suppressed and they change their long-term migratory regimes from superdiffusive to normal diffusion. This suggest that the signalling gradients are spatially smoothed and cell migration progressively becomes Brownian. Also, at short times, the subdiffusive motion of less motile cells becomes even slower. In turn, cells with higher motility seems to be less affected by a varying plated density, although their superdiffusion is enhanced. We hypothesize that, as the density increases, a more ballistic migration reflects the increasing number of such motile cells engaging into large turns generated by longer reorientation flights. (See Figures 1 and 6 in the manuscript.) Again, an increasing homogeneity in cell migratory behavior is observed.

Finally, we analysis the possible nature of the mean squared displacements at short time scales. Active fluctuations of the plasma membrane, lamellipodium protrusions, cell shape variations, and small movements of the cell to probe its surrounding environment comprise the short-term dynamics of cell migration. All these processes alter the cell centroid positions and generate the displacements measured at short-time scales. Different migratory phenotypes can be produced depending on which ones of those aforementioned processes become dominant.

In our assays, the more motile epithelial cells majoritarily exhibt significant shape fluctuations and stronger membrane activity associated to faster, larger and more persistent lamellipodium protrusions. At short time scales this activity seems to be random and practically unaffected by the density of plated cells generating a Brownian motion of the cell centroid. In contrast, in less motile cells, shape fluctuations and membrane activity become much more weak. Also, smaller and less stable lamelipodia are protrused, which are more frequently retracted, generating apparent reversions of the cell centroid motion. The overall result is a subdiffusive cell migration at short time scales. Additionally, as the density of plated cells increases, smoothed signalling gradients impairs even further the activity of less motile cells, getting worse their short-term subdiffusive migration.

4 Turn angle distributions

The turn angle distributions for B16F10 cells at distinct plated densities are illustrated in Figure 3. These distributions are "ω-shaped" curves with local maximums at small (around $0°$) and large angles (around $\pm 180°$). This shape suggest a bimodal analysis in which every individual cell track is subdivided into directional and reorientation "flights". The former (latter) are comprised of successive displacements whose turn angles are always smaller (greater) than a fixed threshold. The results of this analysis are reported in the manuscript.

From Figure 3 one can see that, despite the preferences for small and large turns, the intermediate turn angles have significant probabilities. However, as the cell density increases, the

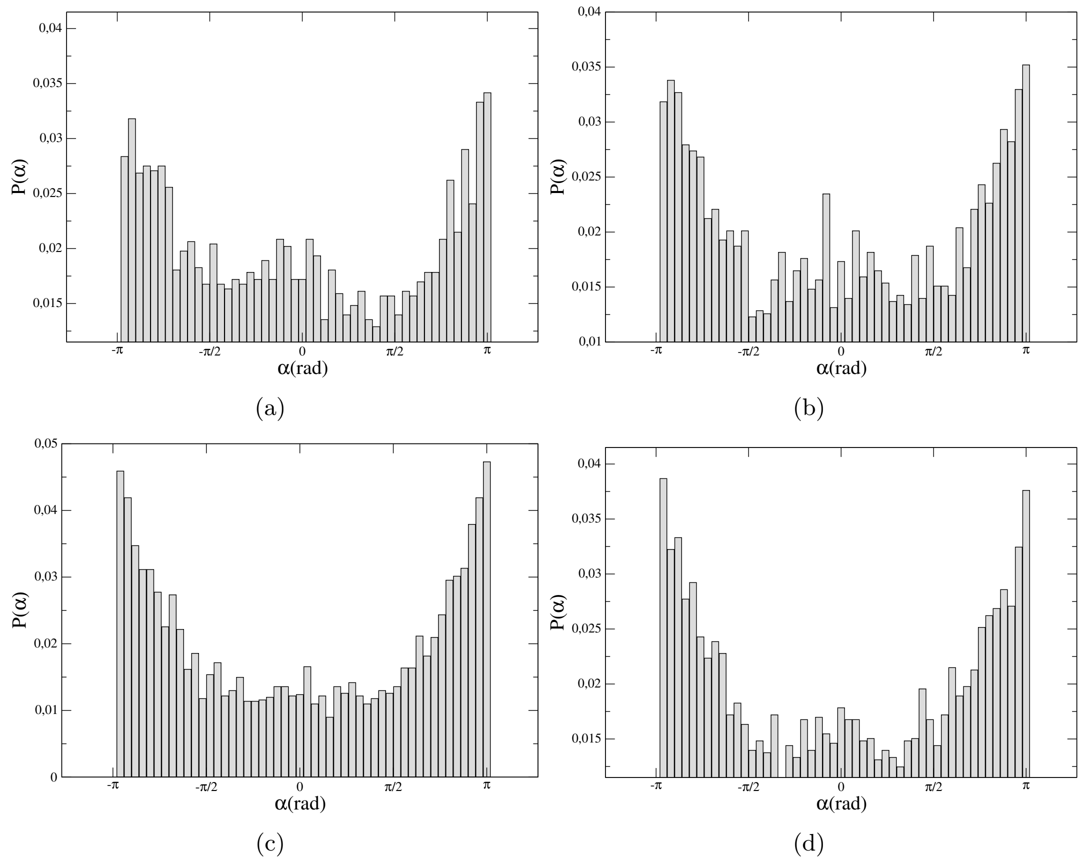

Figure 3: Turn angle distributions for B16F10 cells plated at (a) 20, (b) 100, (c) 2000, and (d) 10000 cells per cm^2. The instantaneous turn angle was defined as the difference between successive displacement orientations for each 1 min time-lapse of each cell's trajectory.

frequencies of small turn angles decrease, although non-monotonically. Hence, the occurence and lenght of directional flights decrease as the cell density increases. On the contrary, it is expected that the occurence and lenght of reorientation flights increase. These results are consistent with a Brownian cell migration pattern at very large plated densities, as indicate the trend observed for the cell speed distributions.
