# Supplementary figures and images for: Anomalous diffusion and q-Weibull velocity distributions in epithelial cell migration
Source: PLoS One. 2017 Jul 10;12(7):e0180777. doi: 10.1371/journal.pone.0180777 (PMC5507264; doi:10.1371/journal.pone.0180777)

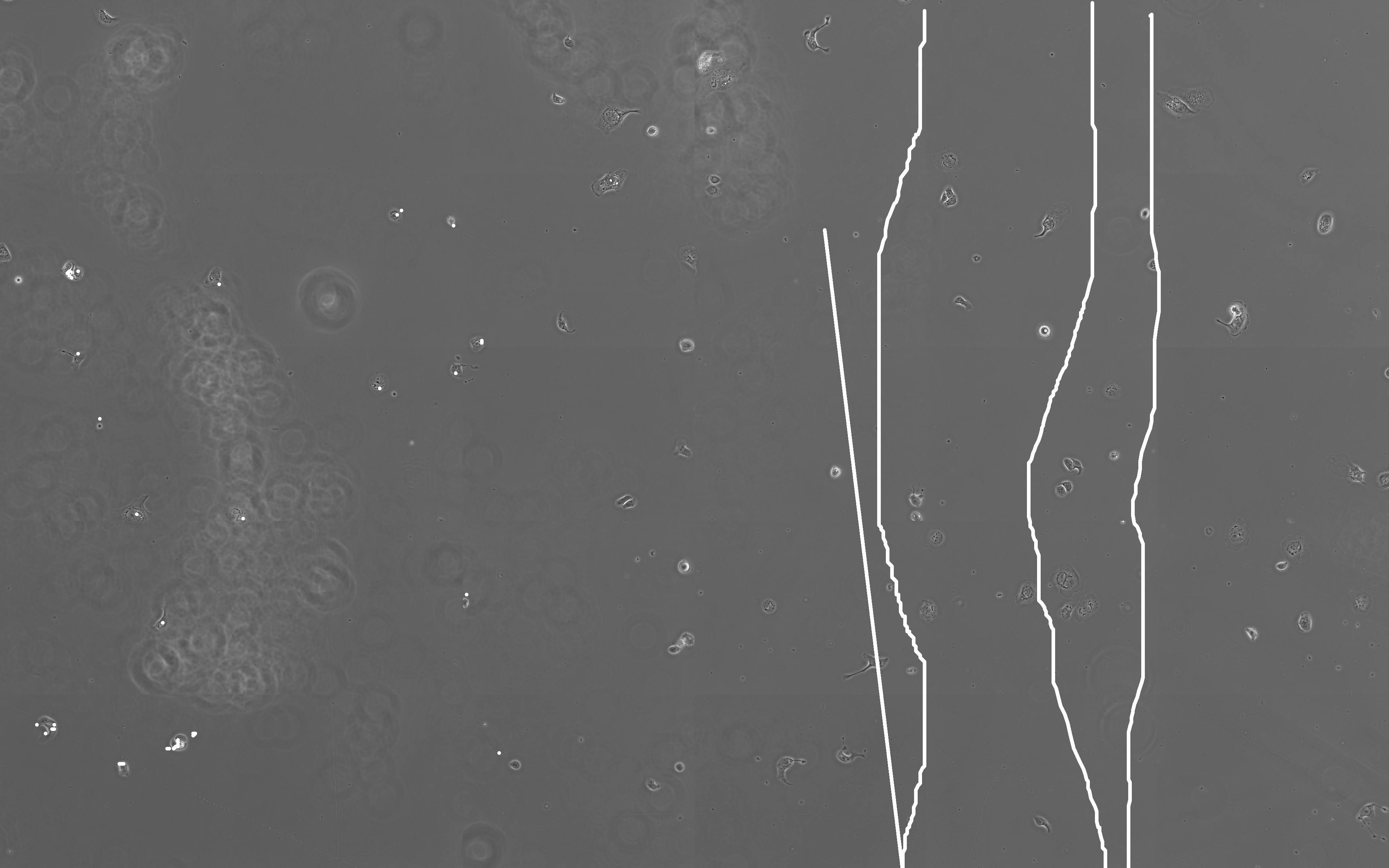

Supplement: S5 File — Individual trajectories of each cell over time for density of 1190 cel/cm2. (available in http://www.posfisicaaplicada.ufv.br/?pageid=2714). (ZIP) [file pone.0180777.s006.zip › d=01190cells/Poco2-ref.tif]

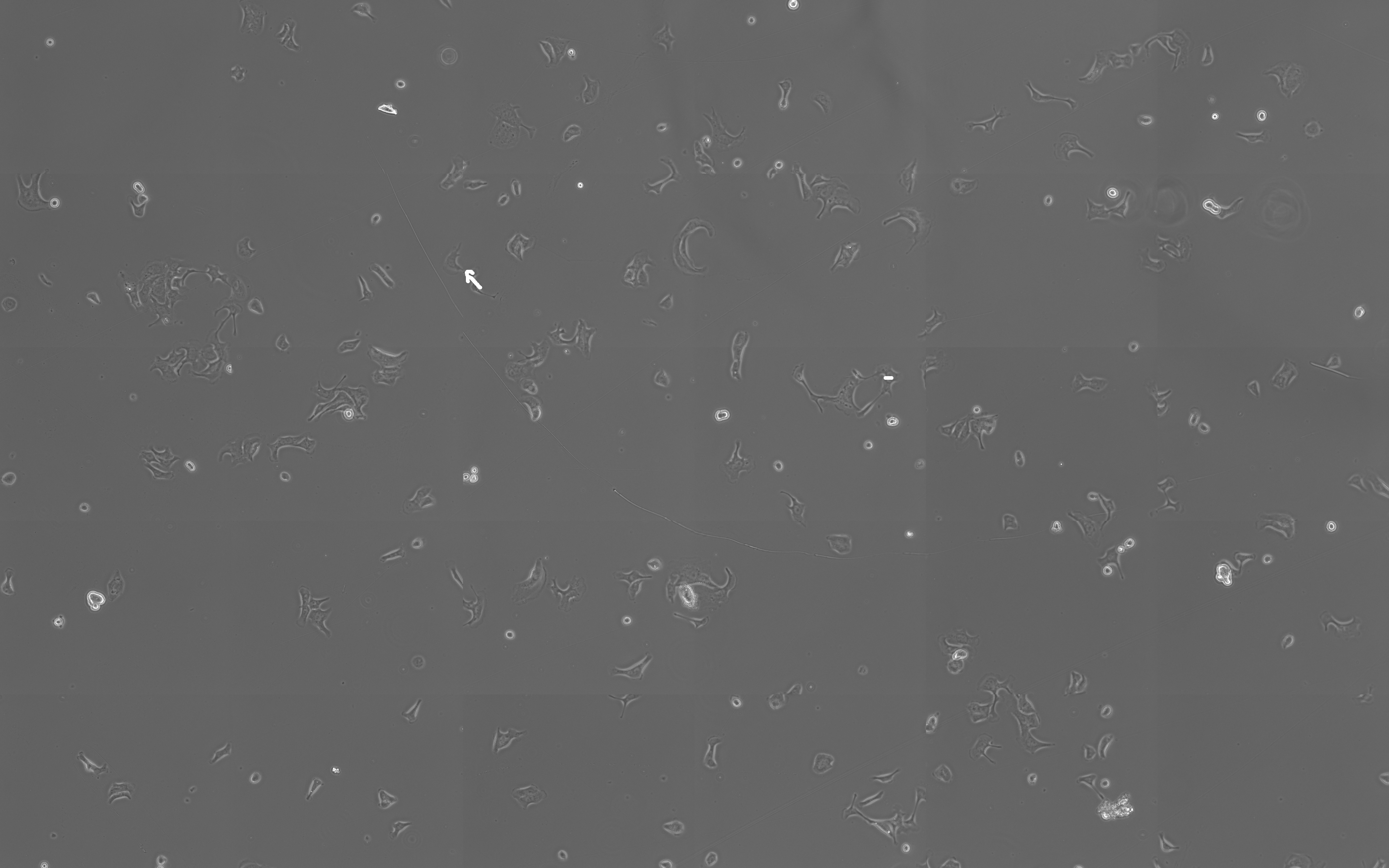

Supplement: S6 File — Individual trajectories of each cell over time for density of 11900 cel/cm2. (available in http://www.posfisicaaplicada.ufv.br/?pageid=2714). (ZIP) [file pone.0180777.s007.zip › d=11900cells/Referencia.tif]
